# Two Cases of Systemic Lupus Erythematosus With Castleman Disease

**DOI:** 10.7759/cureus.102719

**Published:** 2026-01-31

**Authors:** Ana M Bravo-Andrade, Jaime A Ibarra-Burgos, Juliana M Bacca-Gonzalez, Jorge A Torres-Trujillo

**Affiliations:** 1 Hospital Medicine, Clinica Somer, Rionegro, COL; 2 Internal Medicine, Clinica Somer, Rionegro, COL; 3 Pathology, Clinica Somer, Rionegro, COL; 4 Rheumatology, Clinica Somer, Rionegro, COL

**Keywords:** case report, castleman disease, lymphadenopathy, plasma cell variant, systemic lupus erythematosus

## Abstract

Systemic lupus erythematosus is a heterogeneous autoimmune disease that commonly presents with lymphadenopathy, a feature that can lead to confusion with other diseases due to its non-specificity. Histopathological features are usually nonspecific and may mimic other conditions. Other cases have described Castleman disease, a rare lymphoproliferative disorder, in patients with systemic lupus erythematosus. This uncommon association poses diagnostic challenges due to the clinical overlap. We present cases of two Latin-American women with systemic lupus erythematosus and generalized lymphadenopathy, with histopathological findings compatible with plasma cell-subtype Castleman disease, who showed adequate response to rituximab treatment.

## Introduction

Lymphadenopathy is a frequent finding in systemic lupus erythematosus (SLE), occurring in up to 60% of cases at some point during the course of the disease. However, it is a nonspecific manifestation that can be mistaken for other autoimmune, infectious or hematological disorders [1]. Histopathologically, SLE lymphadenopathy is characterized by nonspecific follicular hyperplasia and occasionally, coagulative necrosis. These findings often receive little attention due to their lack of specificity [2-4]. Some studies have identified a possible association between SLE and Castleman disease (CD), a rare lymphoproliferative disorder that can be confused with SLE [5] or occur concomitantly [2-4,6-12]. Histologically, there are two variants of CD: hyaline vascular and plasmacytic. It presents in two clinical forms: unicentric (UCD; affecting a single lymph node or one nodal region) and multicentric (MCD; affecting more than two nodal regions by tomography). In terms of etiology, CD can be idiopathic, or associated with viral infections such as Human Herpesvirus 8 (HHV-8) and Epstein-Barr Virus (EBV) as well as with autoimmune disorders and malignancy [13]. MCD is most frequently associated with autoimmune conditions and immunosuppressed states [14,15]. MCD is more heterogeneous than UCD, and has higher rates of disease progression and lymphoma development, which imposes greater morbidity and mortality; hence, it is clinically important [16]. From a molecular perspective, SLE and CD share some overlapping features of immune dysregulation, but they are driven by different molecular indicators within each entity. SLE is mainly driven by type I interferon production, complement deficiencies, and T cells that contribute to impaired B cell regulation leading to autoantibodies like anti-dsDNA [1]. Idiopathic MCD centers on excessive Interleukin-6 (IL-6) and related cytokines, as well as the mechanistic target of rapamycin (mTOR) pathway activation in monocytes, T cells, and lymph nodes [13,14,17]. Despite having different molecular pathways, overlapping cases pose a diagnostic challenge through shared clinical symptoms and biomarker ambiguity, requiring lymph node biopsy with expert pathology review for diagnostic confirmation. 

There have been very few reports describing lymphadenopathy with CD morphology in patients with SLE. To our knowledge, fewer than 30 cases have been reported in literature to date. In this article, we present two cases of female patients with SLE who presented with lymphadenopathy and had histopathological features compatible with plasmacytic CD.

## Case presentation

Case one

A 20-year-old female patient was admitted with a three-month history of asthenia, adynamia, fever, nocturnal diaphoresis, weight loss of 20 kg and cervical, axillary, and inguinal lymphadenopathy, which had become painful eight days prior to admission. Additionally, she had metacarpophalangeal and proximal interphalangeal arthralgia. She had a history of SLE, which was diagnosed two years prior to the onset of symptoms. Initially, she presented with class IV lupus nephritis, for which she had received multiple immunosuppressive regimens with cyclophosphamide, mycophenolate, azathioprine, hydroxychloroquine, and prednisolone. There was no family history of autoimmune disorders.

Laboratory evaluation showed anemia, leukocytosis due to neutrophilia, thrombocytosis, elevated C-reactive protein (CRP) and erythrocyte sedimentation rate (ESR), proteinuria, no hematuria, and preserved renal and hepatic function (Table 1).

**Table 1:** Laboratory findings at the time of presentation ESR: erythrocyte sedimentation rate; CRP: C-reactive protein; ANA: antinuclear antibodies; anti-DNA: anti-DNA antibodies; ENA: anti-extractable nuclear antigen antibodies; anti-LA: anti-Sjögren’s syndrome type B antibodies; anti-Ro: anti-Ro antibodies; ACA: anti-cardiolipin antibodies; anti-B2-GPI: anti-beta-2 glycoprotein I antibodies.

Lab features	Case 1	Case 2
Haemoglobin (g/dl)	7.7	10.7
White blood cells (nx10^3^/mm^3^)	15.7	3.2
Platelets (nx10^3^/mm^3^)	999	19
ESG (mm/h)	84	49
CRP (mg/L)	139	138
Albumin (g/dl)	2.5	3
Creatinine (mg/dl)	0.54	0.5
Proteinuria (mg/day)	382.8	481.0
C3 (mg/dl)	128	84.8
C4 (mg/dl)	21.8	10.8
ANA	1:1280	1:2560
Anti-DNA	negative	negative
Anti-ENA	negative	anti-LA, anti-Ro
Coombs test	positive	positive
ACA- IgG	positive	not measured
ACA - IgM	negative	not measured
Anti-B2-GPI	negative	not measured
Cryoglobulins	negative	negative

**Figure 1:**
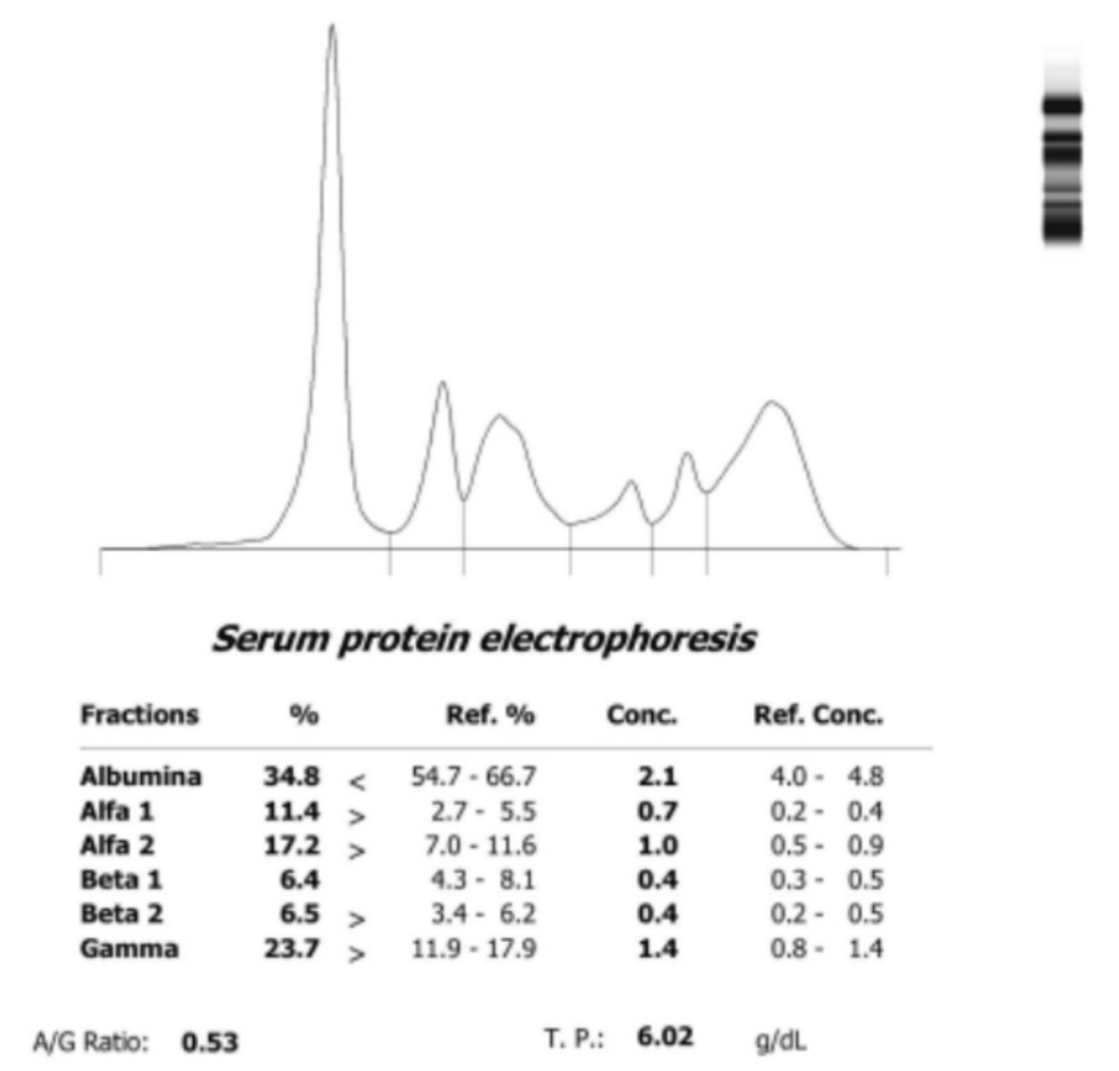
Increase in the alpha 1, alpha 2, beta, and gamma fractions of serum proteins

The autoimmune panel showed antinuclear antibodies (ANA) at a titer of 1:1280 with nuclear patterns Anti-Cell (AC)-1, AC-14, AC-29, but negative anti-extractable nuclear antigens (ENAS) and anti-dsDNA, unconsumed C3 and C4 complement, negative cryoglobulins, antiphospholipid syndrome panel positive with elevated beta 2 microglobulin, and serum protein electrophoresis with polyclonal hypergammaglobulinemia (Figure 1). 

**Figure 2:**
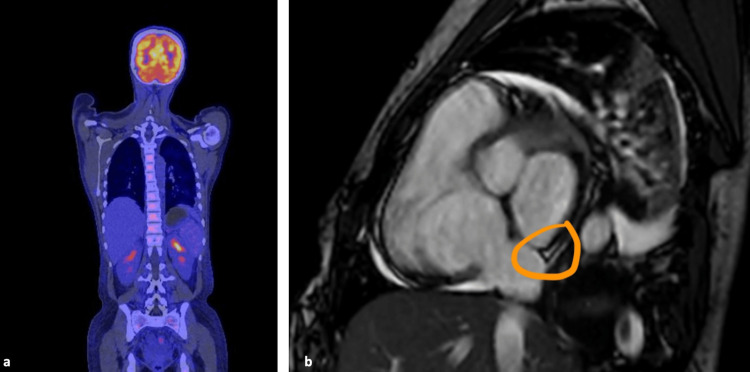
Positron emission tomography CT scan (PET-CT) images a) Bilateral retroperitoneal and pelvic adenopathies with mild increase in metabolism; b) Late linear enhancement in the subepicardial band in the basal and mesial inferolateral segments and lateral apical segment. Myocardium with enhancement =1% of the end-diastolic mass.

Several differential diagnoses were ruled out, including infectious diseases such as: EBV, cytomegalovirus (CMV), tuberculosis, hepatitis B and C, HIV, syphilis, HHV-8 and toxoplasmosis. Blood and urine cultures were negative. Imaging studies included chest and abdomen computed tomography (CT scan), which showed ground-glass opacities in the lower left lobe. Hepatomegaly and inguinal, iliac, and retroperitoneal lymphadenopathies were also found on positron emission tomography CT scan (PET-CT) (Figure 2a).

Inguinal lymph node biopsy was performed, and histological findings of plasmacytic CD were observed. Molecular panel from the bronchoalveolar lavage (BAL) identified *Haemophilus influenzae* and *Staphylococcus aureus*, both were treated. Magnetic resonance imaging (MRI) of the heart performed to investigate chest pain, revealed acute myocarditis (Lake Louise revised criteria 2/2 [18]) with very poor late enhancement (Figure 2b). Finally, rheumatology considered severe lupus activity with joint, myocardial, and hematolymphoid involvement, for which the patient received treatment with pulses of methylprednisolone, rituximab, and azathioprine, with clinical improvement.

Case two

A 36-year-old woman of indigenous ethnicity who was admitted in late postpartum, presented with fever and proximal interphalangeal arthralgia. She had a history of SLE, newly diagnosed at 34+1 weeks of pregnancy, which manifested as bicytopenia, arthralgia, adenopathy, and fever. She was treated with hydroxychloroquine and prednisolone for this condition and had a vaginal delivery at 34+3 weeks. Prior to this pregnancy, she had no relevant medical history. 

**Figure 3:**
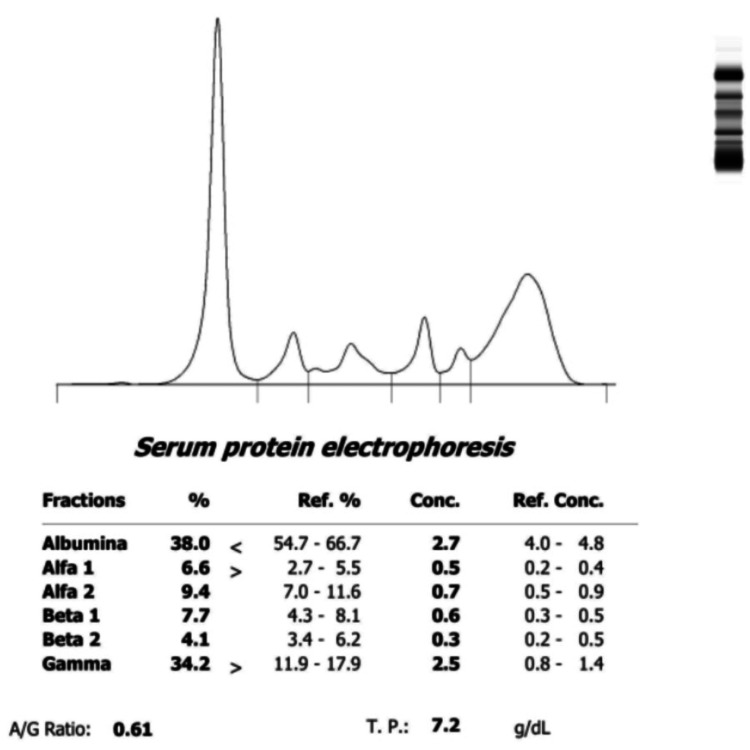
Increase in the alpha 1 and gamma fractions of serum proteins

Physical examination revealed cervical and axillary lymphadenopathy, as well as oral candidiasis, which was treated. Laboratory tests revealed autoimmune hemolytic anemia, leukopenia, thrombocytopenia, decreased C3 complement, hematuria with proteinuria, and elevated ESR and CRP (Table 1). The autoimmune panel reported ANAs at titers of 1:2560 with AC-1, AC-4, AC-14, AC-29, anti-Sjögren’s syndrome type B antibodies (anti-SSB LA), and Ro positive nuclear patterns (both greater than 200 U/ml), anti-Smith (anti-SM) and Ribonucleoprotein (RNP) negative, cryoglobulins negative, beta 2 microglobulin positive, and protein electrophoresis with polyclonal hypergammaglobulinemia (Figure 3).

**Figure 4:**
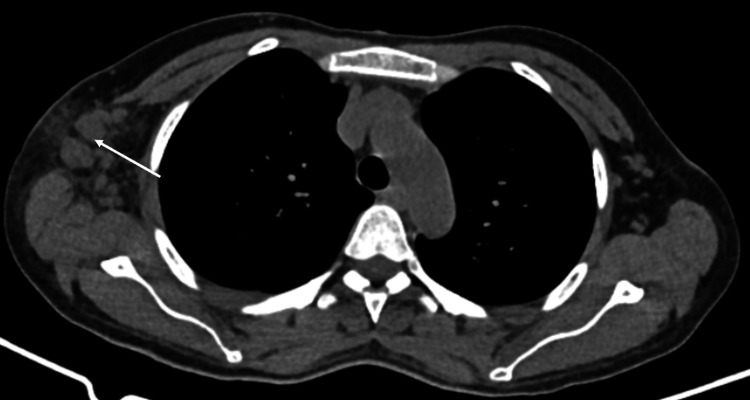
The white arrow indicates an increase in the number and size of axillary lymph nodes on the right side, the largest of which measured 26x32 mm

Infectious etiologies were considered in the differential diagnosis but were ultimately ruled out: EBV, CMV, tuberculosis, HIV, syphilis, dengue, toxoplasmosis, leptospirosis, malaria, hepatitis B, and C. Urine and blood cultures were negative. Imaging studies were performed, including chest and abdominal CT scans, which showed axillary and mediastinal adenomegaly as well as splenomegaly. PET-CT imaging was performed to complement the evaluation and led to the decision to biopsy the axillary lymph nodes, which reported morphological and phenotypic findings consistent with plasmacytic subtype CD (Figure 4).

Finally, rituximab was administered, resulting in clinical improvement. 

Histopathological findings

**Figure 5:**
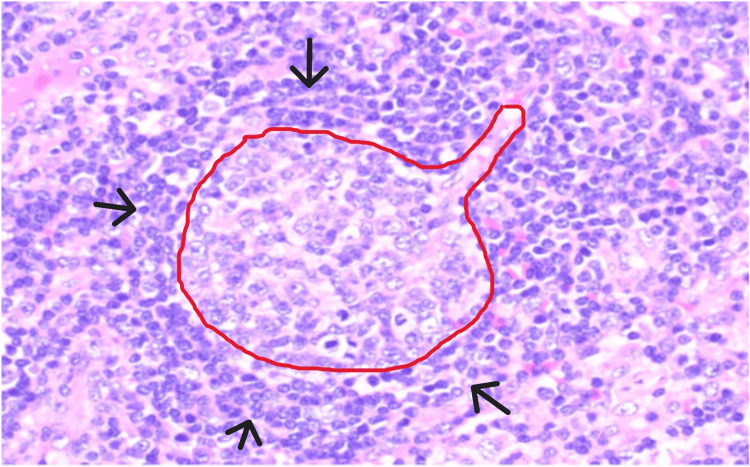
Histopathological image Radially-oriented and hyalinized blood vessels penetrating the germinal centers, forming hyaline vascular lesions (lollipop morphology). The mantle zone expands with lymphocytes arranged concentrically in a ring (onion skin morphology). Lymphocyte-depleted germinal centers and paracortical vascular hyperplasia are also seen.

In both cases, histopathological findings consistent with CD were found, with a general lymph node architecture distorted due to follicular and paracortical hyperplasia [14,15]. Specifically, the plasmacytoid/mixed subtype, in which hyperplasia is accompanied by follicles that are depleted of lymphocytes and enriched with follicular dendritic cells. Radially-oriented, hyalinized or sclerotic blood vessels had penetrated the germinal centers, forming lollipop-shaped hyaline vascular lesions. Lymphocytes expanded the mantle zones and were arranged in concentric onion-skin-like rings around the germinal centers (Figure 5).

**Figure 6:**
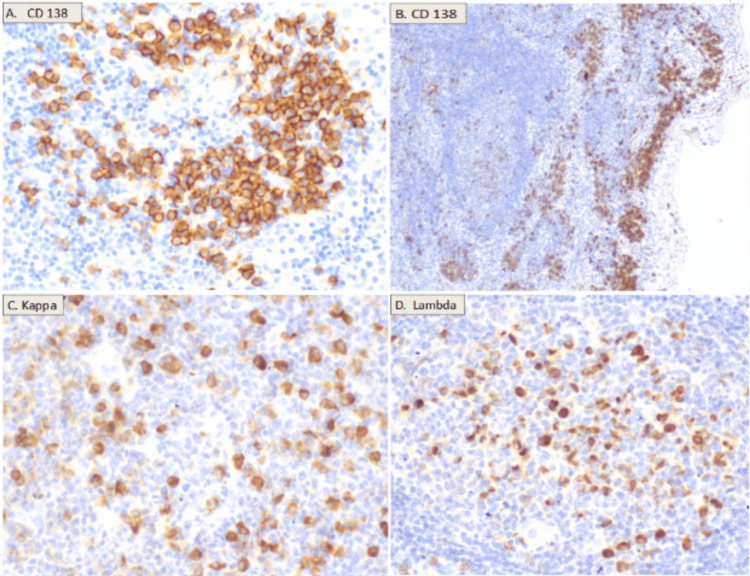
Histopathological images Interfollicular (A) and subcapsular (B) plasmacytosis with dense clusters of mature CD138+ plasma cells, polytypic with a kappa/lambda (K/L) ratio of 1, preserved ratio (C,D).

Interfollicular areas consisted of small, mature lymphocytes without atypia and contained numerous high-endothelial venules, many of which were sclerotic. There was also an increase in mature and polytypic plasma cells for light chains (Figure 6), distributed in dense paracortical and subcortical groups with a preserved IgG4/IgG ratio of less than 40%. 

The sinusoids appeared collapsed and the capsule was fibrous and thickened. 

The immunohistochemical findings in both cases were similar, ruling out lymphomas, IgG4 disease, plasmacytoma, infections, and association with human herpesvirus type 8 and EBV. In each case, the following panel was performed: Cluster of differentiation 20 (CD20), Cluster of Differentiation 3 (CD3), Cluster of Differentiation 30 (CD30), Ki67, Cluster of Differentiation 23 (CD23), Cluster of Differentiation 138 (CD138), kappa, lambda, IgG4, IgG, Latent Membrane Protein 1 of the Epstein-Barr Virus (LMP1-EBV), Latency-Associated Nuclear Antigen of Human Herpesvirus 8 (HHV-8-LANA), Cluster of Differentiation 10 or Common acute lymphoblastic leukemia antigen (CD10), B-Cell Lymphoma 2 (BCL2), B-Cell Lymphoma 6 (BCL6), Cluster of Differentiation 5 (CD5), Cluster of Differentiation 43 (CD43), Cyclin D1 (CCND1), and SRY-Box transcription factor 11 (SOX11), according to the recommendations established by the Spanish Society of Pathological Anatomy for the study of differential diagnoses in all cases of CD [19].

## Discussion

We present two cases of women of reproductive age who presented with fever and lymphadenopathy in different lymph node stations. In case one, palpitations, dyspnea, and chest pain were documented, while in case two, polyarthralgia was evident. Both patients presented with hematological compromise due to hemolytic anemia. However, the behavior of the other cell lines differed: leukocytosis and thrombocytosis were observed in the first case, while leukopenia and thrombocytopenia were observed in the second. This could be explained by the different inflammatory drivers of SLE and CD. Thrombocytosis is frequent in CD due to IL-6 pathways, MCD often shows thrombocytopenia from hypersplenism or consumption, while in SLE, it stems from autoimmune destruction [13,17]. This report highlights the diagnostic convergence of both cases, given the opportunity to perform a histological analysis of the lymphadenopathies. Surprisingly, both cases exhibited a morphology consistent with MCD, plasmacytic/mixed subtype.

Similar demographic characteristics have been reported in other studies, with most being female patients between the ages of 16 and 44. Manifestations were similar to those reported in our cases [2,3,6,7,11,12], although most of them had not been previously diagnosed with SLE, they met the Systemic Lupus International Collaborating Clinics (SLICC) classification criteria and were diagnosed with the disease [2-4,6-10]. Additionally, the majority of previously reported cases correspond to Asian patients [2-4,7-12]. To our knowledge, this is the first report of these two conditions in Latin America. 

While the follicular hyperplasia observed in both of our cases favors SLE, it remains non-specific. However, when paired with CD-specific vascular features such as lollipop morphology, it shifts the diagnosis toward CD [5,13,17]. Most previously reported cases of SLE with CD have been multicentric, only one report was unicentric [2], and a slight predominance of the plasmacytic histological subtype [4,6,8,20,21] over the vascular hyaline subtype [3,4,7,9-11,20] has been observed, which is consistent with the findings at our institution. 

The diagnosis and management of CD represents a clinical challenge because it is a rare disease and its symptoms are very heterogeneous, ranging from asymptomatic lymphadenopathy with or without chronic inflammation, to a severe and rapidly progressive cytokine storm. In addition, the histological characteristics of CD are not specific to this disease and can be observed in infectious, autoimmune, and neoplastic diseases [13]. In our cases, infections and neoplasia were ruled out and, given the findings, idiopathic MCD with a clinical subtype not otherwise specified (NOS) could be considered; however, the presence of SLE must take precedence. 

Treatment is generally based on the use of corticosteroids, and some cases also receive rituximab [7,8], chemotherapy [6-8], or immunosuppressants [6,8,10]. Patient mortality was reported in two cases despite pharmacological management [6,8]. In our cases, both patients received almost a month of steroid therapy without remission. Rituximab was then administered, and the short-term outcome was favorable, with resolution of symptoms and fulfillment of the criteria for hospital discharge. Unfortunately, long-term follow-up was not possible for either of the cases presented.

## Conclusions

These cases highlight the diagnostic complexity with the possibility of clinical and histological overlap between both pathologies. Although the presence of SLE is an exclusion criterion in the diagnostic criteria for CD, as outlined in the literature above, some reports have documented the coexistence of both conditions, challenging this distinction. There is a need for modern immunohistochemical studies to correlate these findings with the morphological changes observed in both diseases.

In conclusion, lymphadenopathy, as a common and nonspecific manifestation of SLE, should always prompt a thorough evaluation, ideally by hematopathology. However, differentiating it from conditions such as CD presents a challenge based on current knowledge. Each patient requires an individualized therapeutic approach, where SLE should take precedence. Physicians should also aim to report similar cases and generate knowledge about the coexistence of these entities, leading to new treatments and improved outcomes.
